# Efficient Regeneration and Genetic Transformation System for Cymbidium kanran ‘Zhushalan’

**DOI:** 10.3390/genes17050515

**Published:** 2026-04-27

**Authors:** Hua Cao, Bin Zhou, Lin Lu, Yuying Zhang, Guanghong Li, Shenchong Li, Han Li

**Affiliations:** 1 Flower Research Institute, National Engineering Research Center for Ornamental Horticulture, Yunnan Academy of Agricultural Sciences, Kunming 650205, China; 2 College of Landscape and Horticulture, Yunnan Agricultural University, Kunming 650201, China; 3 Zhouning County Forestry (Flower) Industry Development Center, 2nd Floor, Shicheng International, Shicheng Town, Zhouning County, Ningde 355400, China; 4 Dali Orchid Industry Development Co., Ltd., Dali 671000, China

**Keywords:** *Cymbidium kanran*, genetic transformation, regeneration system, orchid breeding, rhizom, Agrobacterium-mediated transformation

## Abstract

Background: *Cymbidium kanran* ‘Zhushalan’ is a famous traditional Chinese orchid with high ornamental and economic value. As its market expands, there is a need to improve its key horticultural traits and stress resistance. Unfortunately, these traits are difficult to breed using traditional methods, and an optimal regeneration and genetic transformation system for *C. kanran* has yet to be established. Methods: This study evaluated the factors affecting Agrobacterium-mediated genetic transformation and regeneration of *C. kanran* ‘Zhushalan’ using rhizomes obtained from seedlings as receptor material. Results: The highest regeneration frequency was achieved after pre-cultivating the rhizomes in the dark on ½ MS medium for 10 days. The genetic transformation system was optimized as follows: Agrobacterium strain, EHA105; optimal concentration of Agrobacterium solution, OD_600_ = 0.6; 100 mg·L^−1^ acetosyringone; an infection time of no more than 40 min; and co-culturing for one to three days. Positive strains were screened using meropenem (15 mg·L^−1^) and hygromycin (50 mg·L^−1^) and confirmed through PCR and qRT-PCR. A transformation rate of 11.67% was achieved. Conclusions: An efficient regeneration and genetic transformation system for *C. kanran* ‘Zhushalan’ has been established for developing transgenic technologies. Our findings will stimulate research on functional genes and molecular breeding related to *C. kanran.*

## 1. Introduction

*C. kanran* is one of the seven major species of Chinese orchids. It is mainly distributed in Fujian, Taiwan, Guangdong, Guangxi, and Yunnan, among other regions of China. Its flowering period ranges from October to January of the following year. *C*. *kanran* ‘Zhushalan’ is a precious traditional cultivar and a representative variety of *C. kanran* in Yunnan Province. It has a long flowering period, high ornamental and economic value, and has been highly sought after by people since ancient times. Therefore, there is a need to develop tools and strategies to sustain and improve its production. Unfortunately, the production and market of Chinese orchids still face many challenges, such as long flowering cycles, unclear molecular regulation of flowering periods, limited flower types and colors, severe diseases, and environmental stresses [1,2]. Introducing new traits in orchids, such as new flower colors, through mutation breeding or hybrid breeding is difficult. However, transgenic technology can easily achieve targeted modification of orchid traits [3,4].

Transgenic technology is an important means of improving desirable plant traits through genetic engineering. Since the first genetically modified plants were obtained in 1983, research on plant genetic transformation has advanced rapidly [5]. Transgenic technology transfers the desired target genes into plants and can programmatically inhibit or enhance gene expression. This technology has been used to enhance the ornamental value of horticultural plants by modifying their genomes [6]. Compared with traditional breeding strategies, transgenic methods have greater potential for creating new phenotypes. Currently reported plant genetic transformation methods include Agrobacterium-mediated transformation, particle bombardment, and the pollen tube pathway. Among them, Agrobacterium-mediated transformation and particle bombardment are the most commonly used methods in orchid breeding [7]. Agrobacterium-mediated transformation is characterized by high efficiency and low cost and is widely used for the stable genetic transformation of plant species [8,9]. This method was successfully used for the genetic transformation of Phalaenopsis orchids in 1997 [10], and *Dendrobium sonia* in 1998 [11,12]. Recent advances in molecular biotechnology have led to great achievements in the genetic transformation of other orchids, including *Dendrobium Sonia* ‘Earsakul’ [13], *Dendrobium* ‘Chao Praya Smile’ [14], *Erycina pusilla* [15], *Oncidium* [16], *Phalaenopsis* [17], and *C*. *sinense* [18]. These advances have resulted in improvements in key horticultural plant traits, including flower color [19], floral fragrance [20,21], flower development [22,23], and disease resistance [24]. In addition, advances in genomics, bioinformatics, and sequencing technologies have led to the publication of the whole genome data of orchids, such as *Phalaenopsis aphrodite* [25], *C*. *goeringii* [26], and *C*. *ensifolium* [27], laying a solid foundation for transgenic breeding of orchids.

The selection of genetic transformation receptor materials and their regenerative ability are crucial for efficient transformation. The receptor material must have efficient and stable regeneration capabilities, high genetic stability, and a large number of stable explant sources [1]. Callus tissue is commonly used as a receptor material for genetic transformation in orchids. However, it is generally believed that callus tissue from Orchidaceae plants is not a suitable transformation receptor because of the difficulty in maintaining single cells. In particular, callus tissue is prone to mutation and the formation of chimeras. Kuehte et al. argued that embryogenic callus is a more suitable transformation receptor material for orchids because it can be obtained directly from regenerated plants after transformation. This eliminates the need for embryonic development, which makes callus chimeras and new somatic embryos difficult to form. Additionally, non-transformed cells can be effectively removed during this process [28]. However, inducing embryogenic callus in orchids requires adding special substances, and the induced callus tissue has limited growth ability, making subculture difficult. Protocorms, a unique growth state of a few plants in Orchidaceae, are easy to induce and have a very high instantaneous expression rate after co-cultivation. Inducing callus tissue allows regeneration into protocorm-like bodies (PLBs), which can then regenerate whole plants [29]. Therefore, protocorms and PLBs are currently ideal transformation receptor materials for orchids, with high regeneration rates in transformation. PLBs have been successfully used as transformation receptors in *P. aphrodite*, *D. nobile*, *O. sphacelatum*, and *D. officinale* [12,30,31]. In *Cymbidium*, due to the special shape of its protocorms and PLBs, they are referred to as rhizomes. Apart from callus tissue and protocorms, other receptor materials for orchid genetic transformation have been reported, including suspended cells, pollen, and immature embryos [10,32,33,34]. In addition to the transformation receptor, several other factors affect the success and efficiency of orchid genetic transformation, such as the pre-culture time [35], Agrobacterium strain [12], concentration of the bacterial solution [36,37], infection mode and time [10], and co-culture time [38].

Currently, the genetic transformation of most Orchidaceae species is still at the preliminary exploration stage. Only a few species, such as *P. aphrodite*, *D. nobile*, and *O. sphacelatum,* have relatively well-established genetic transformation systems. *Cymbidium* plants are hindered by rhizome induction. However, the genetic transformation system of only one species, *C. sinense,* has been reported due to the advanced technology of inducing rhizomes through lateral buds. In short, many highly favored orchids, such as most Chinese orchids, including *C. kanran,* have not achieved key breakthroughs in genetic transformation research. Particularly, a comprehensive and systematic analysis of the factors affecting the transformation frequency in *C. kanran* has yet to be conducted. Due to the high heterogeneity among Chinese orchids, the transformation systems established for other Cymbidium species are not expected to produce similarly efficient results in *C. kanran*. Therefore, establishing an efficient genetic transformation system for *C. kanran* ‘Zhushalan’ is crucial. This will advance research on functional genes and facilitate the molecular-assisted breeding of important traits.

In this study, we used rhizomes as receptor materials and evaluated the effects of key factors such as pre-culture time, bacterial solution concentration, infection time and method, and co-culture time on the regeneration and transformation rate of *C. kanran* ‘Zhushalan’ rhizomes. Our goal was to develop an efficient genetic transformation and regeneration system for *C. kanran* ‘Zhushalan’. The results of this study lay the groundwork for validating functional genes underlying key agronomic traits in *C. kanran* and for further developing genomics-assisted breeding in Cymbidium.

## 2. Materials and Methods

### 2.1. Plant and Plasmid Materials

**Figure 1:**
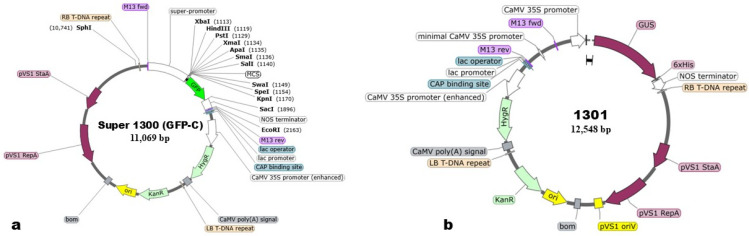
The plasmid profiles of pCAMBIA1300-GFP (**a**) and pCAMBIA1301-GUS (**b**).

The *C. kanran* ‘Zhushalan’ seeds were collected from Dali Languo Flower Industry Development Co., Ltd. (Dali, China). All collected seeds were derived from self-pollination and matured for ten months. After sterilization with 75% (*v*/*v*) ethanol for 30 s and 0.1% (*w*/*v*) HgCl2 for five minutes, the seeds were rinsed and then aseptically sown in the germination medium (Table 1). After 180 days, healthy rhizomes with uniform growth (1–2 cm) were selected and transferred onto the propagation medium (Table 1) to obtain a large number of rhizomes. The plasmids used in the experiment were pCAMBIA1301, which contains the T-DNA region with hygromycin phosphotransferase (HPT) and β-glucuronidase (GUS) genes, and pCAMBIA1300, which contains the T-DNA region with HPT and green fluorescent protein (GFP) genes (Figure 1). All the above materials are stored at the Flower Research Institute of the Yunnan Academy of Agricultural Sciences. All experiments were conducted under aseptic conditions. All factors evaluated, including pre-culture time, Agrobacterium strain, concentration of the bacterial solution, infection mode and time, and co-culture time, were set based on previous studies [12,35,36,37,38].

### 2.2. Culture Media

The culture medium was prepared using ½ MS medium, tryptone, activated carbon, agar, sucrose, 6-benzylaminopurine (6-BA), and naphthaleneacetic acid (1-naphthaleneacetic acid (NAA) at a pH level between 5.8 and 6.0. The composition of the culture media used in this study is presented in Table 1.

### 2.3. Selection of the Agrobacterium Strain

To identify a suitable Agrobacterium strain, the pCAMBIA1301-GUS vector plasmid was introduced into *Agrobacterium tumefaciens* strains LBA4404, EHA105, and GV3101. Each strain was then added to YEB medium and cultured overnight until the OD600 reached 0.8–1.0. Each treatment consisted of 30 rhizomes infected for 60 min and was independently repeated three times.

### 2.4. Genetic Transformation and Regeneration Workflow

The genetic transformation and plant regeneration process followed a systematic sequence: pre-culture, infection, co-culture, selection, regeneration, and molecular confirmation. The detailed workflow is as follows:

Step 1: Pre-culture. Healthy rhizomes (approx. 1 cm) with good growth potential were selected and then subjected to dark cultivation for different periods (5, 10, 15 days) to obtain tender tissues receptive to infection and assess the effect of dark cultivation duration on the transformation frequency and regeneration rate of *C. kanran* ‘Zhushalan’. Each treatment consisted of 60 rhizomes infected for 60 min and was independently repeated three times.

Step 2: Infection. The pre-cultured rhizomes were slightly wounded and submerged in Agrobacterium suspension. Different concentrations, including OD600 of 0.4, 0.6, 0.8, and 1, were evaluated. To identify the optimal infection time and mode for maximizing the regeneration frequency, different shaking (200 r/min) times, including 20 (S20), 40 (S40), and 60 (S60) minutes, were first evaluated, and S40 was identified as the most optimal. Next, three combinations of S40 and vacuum for different times, including S40V10, S40V20, and S40V30, were evaluated. After five days of co-cultivation, GUS histochemical staining was performed to detect and quantify the transient expression rate of GUS. Each treatment consisted of 30 (transformation) or 60 (regeneration) rhizomes, independently repeated three times.

Step 3: Co-culture. After removing excess bacterial solution with sterile filter paper, the rhizomes were transferred to co-culture medium containing acetosyringone (AS) and incubated in the dark for one, three, or five days at 25 °C. Different concentrations of AS, including 0, 100, 200, and 300 μmol·L^−1^, were evaluated, with the treatment of 0 μmol·L^−1^ representing the control. AS, a phenolic compound naturally released by wounded plant cells, induces the virulence (vir) genes of *Agrobacterium tumefaciens*. This induction is crucial for the transfer of T-DNA from the bacterium into the plant cell genome. After five days of co-cultivation, the transient expression rate of GUS was detected via histochemical staining. Each treatment consisted of 30 (transformation) or 60 (regeneration) rhizomes, independently repeated three times.

Step 4: Selection and Regeneration. Following co-culture, the rhizomes were washed with sterile water containing 500 mg/L cefotaxime and transferred to regeneration screening medium (containing 15 mg/L meropenem and 50 mg/L hygromycin). The medium was refreshed every 7 days. These cultures were maintained at 25 °C under a 14 h light/10 h dark photoperiod for 30 days to induce shoot regeneration. The mortality rate was determined after 14 days. Different concentrations of hygromycin (Hgy), including 0, 20, 30, 40, 50, and 60 mg·L^−1^, were evaluated, with 0 mg·L^−1^ of Hgy representing the control. Each treatment consisted of 30 (transformation) or 60 (regeneration) rhizomes, independently repeated three times.

Step 5: Confirmation. Transgenic candidates were initially screened by GFP fluorescence observation. Subsequently, stable integration of the target gene was confirmed in regenerated plants via PCR and qRT-PCR analysis. Three biological and technical replicates were applied for both PCR and qRT-PCR.

To clearly distinguish between the different developmental stages of the transformation process, the following four parameters were calculated using the formulas below [39]:

Transient Expression Rate = (Number of rhizomes showing GFP signals/Total number of infected rhizomes) × 100%

Survival Rate = (Number of surviving rhizomes on selection medium/Total number of infected rhizomes) × 100%

Regeneration Frequency = (Total number of newly formed buds/Number of surviving rhizomes) × 100%

Transformation Efficiency = (Number of PCR-confirmed positive plants/Total number of infected rhizomes) × 100%

### 2.5. Transgenic Plant Identification

#### 2.5.1. Green Fluorescent Protein (GFP) Fluorescence Microscopy Detection

After sterilization, the rhizomes were observed using GFP fluorescence. The fluorescence signal was detected, and the pictures were taken with a Leica MZ16. The number of fluorescing rhizomes in each group was counted, and the fluorescence rate was determined using the above formula.

#### 2.5.2. Molecular Biology Testing

Polymerase chain reaction (PCR) and quantitative reverse transcription PCR (qRT-PCR) are widely used to confirm transgenic lines [39,40]. In this study, transgenic plants were detected using PCR and qRT-PCR with the following primers:

*GFP* gene (411 bp): F: GCCATTTCGCCTTTTCAG and R: GTAGCGCGTGAGACTG.

Internal reference gene (*ACTIN*)*:* F: ATGGCCGACGGTGAAGAAAT and R: GCAAAACCAGCCTTGACC.

### 2.6. Data Processing and Statistical Analysis

Data were statistically analyzed using SPSS 24.0 and Excel 2019. Data are presented as the mean ± SD of all replicates. Statistical differences among treatments were determined via One-Way ANOVA (analysis of variance) with a post hoc Tukey test. Significant differences were set at *p* < 0.05.

## 3. Results

### 3.1. Infectivity of Three Agrobacterium Strains on C. kanran ‘Zhushalan’

**Figure 2:**
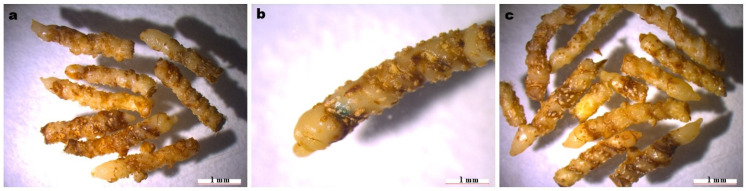
Infection of *C. kanran* ‘Zhushalan’ by different Agrobacterium strains, as revealed by GUS staining: (**a**) LBA4404; (**b**) EHA105; (**c**) GV3101.

Based on the transient expression rate of GUS, EHA105 exhibited the strongest infection capability. Notably, the expression rate of GUS recorded with EHA105 was 16.01-fold higher than that of GV3101 and LBA4404 (Table 2, Figure 2). These results show that EHA105 can be adopted for *C. kanran ‘Zhushalan’* transformation.

### 3.2. Infectivity of Different Concentrations of Acetosyringone (AS) on C. kanran ‘Zhushalan’

**Figure 3:**
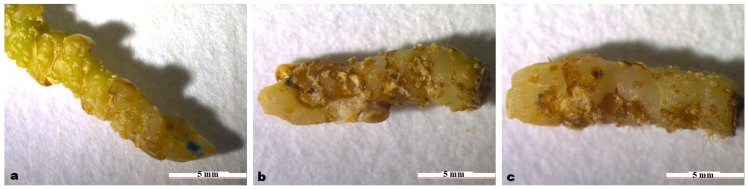
Effects of different concentrations of acetosyringone on *C. kanran* ‘Zhushalan’ infection, as revealed by GUS staining. (**a**) 100 μmol·L^−1^ AS; (**b**) 200 μmol·L^−1^ AS; (**c**) 300 μmol·L^−1^ AS.

AS significantly affects the infection rate of Agrobacterium strains through its induction effect on vir. As illustrated in Table 3, the addition of AS improved the transient expression of GUS in *C. kanran* ‘Zhushalan’. The highest transient expression rate of GUS was reached at an AS concentration of 100 μmol/L. However, as the AS concentration increased, the transient expression rate of GUS decreased significantly, indicating that AS begins to exert an inhibitory effect (Table 3, Figure 3). Thus, the optimal AS concentration for the genetic transformation of *C. kanran* ‘Zhushalan’ was determined to be 100 μmol·L^−1^.

### 3.3. Effect of Hygromycin on the Rhizomes of C. kanran ‘Zhushalan’

Hygromycin is the most commonly used antibiotic for selecting transformed explants. As shown in Table 4, the concentration of hygromycin significantly impacted the growth and survival of *C. kanran* ‘Zhushalan’. The survival rate of rhizomes decreased significantly with increasing hygromycin concentration. The death rate of rhizomes approached 100% when the concentration of hygromycin in the culture medium was 50 mg·L^−1^. Therefore, 50 mg/L of hygromycin was determined to be an appropriate screening concentration.

### 3.4. Factors Affecting the Conversion Efficiency of C. kanran ‘Zhushalan’

#### 3.4.1. Effect of Dark Culture Time on the Conversion Rate of Rhizomes

**Figure 4:**
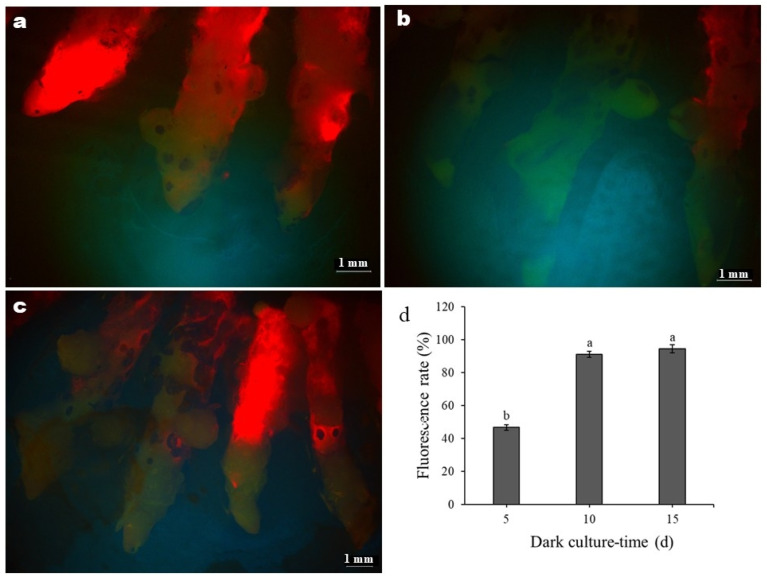
(**a**–**c**) Fluorescence responses of rhizomes after 5, 10, and 15 days of dark culture, respectively. The magnification is 10×. The green part of GFP has been successfully expressed, and the red part of GFP has not been expressed. (**d**) Fluorescence rate at different dark incubation times. Data are shown as mean ± SD. Different letters indicate significant differences between treatments at *p* < 0.05.

**Figure 5:**
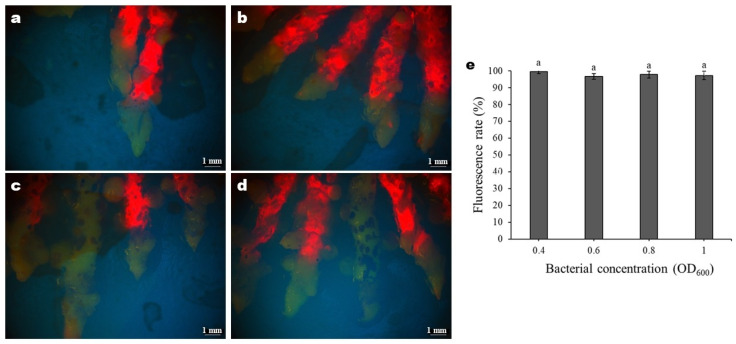
Fluorescence responses of rhizomes under different bacterial concentrations: (**a**) OD_600_ = 0.4; (**b**) OD_600_ = 0.6; (**c**) OD_600_ = 0.8; (**d**) OD_600_ = 1.0. The green part of GFP has been successfully expressed, and the red part of GFP has not been expressed. (**e**) Fluorescence rate under different bacterial concentrations. Data are shown as mean ± SD. Different letters indicate significant differences at *p* < 0.05.

Dark culture is essential for higher genetic transformation efficiency in plants. To determine the optimal pre-culture time in the dark, we observed the fluorescence of GFP in infected rhizomes treated with different pre-culture times (Figure 4). As shown in Figure 5a, the fluorescence response was insignificant. Only a few rhizomes exhibited a weak fluorescence response at the tip after five days of dark cultivation. In contrast, the rhizomes cultured in the dark for 10 or 15 days exhibited stronger fluorescence responses that were highest and statistically identical (Figure 5b,c). Thus, further statistics were conducted on the number of rhizomes that exhibited fluorescence under different dark culture times. The results showed significant differences in the fluorescence rate of rhizomes relative to dark culture times, with the lowest rate recorded after five days (Figure 4d). Notably, the fluorescence rate of the rhizomes was less than 50% after five days of dark pre-cultivation, whereas it significantly increased to 90% for 10 or 15 days (Figure 4d). Therefore, from the perspective of fluorescence rate, the optimal dark pre-culture time for genetic transformation of *C. kanran* ‘Zhushalan’ is set to be 10 or 15 days.

#### 3.4.2. Effect of Bacterial Concentration on the Conversion Rate of Rhizomes

The concentration of the bacterial solution significantly affects the transformation frequency of plants. As shown in Figure 5e, the concentration of the bacterial infection solution did not significantly affect the fluorescence rate. Most rhizomes only exhibited a strong fluorescence reaction in the tender part of the stem tip. Only when the Agrobacterium solution OD_600_ concentration was 0.8 or 1.0 did some rhizomes exhibit a better fluorescence reaction (Figure 5a–d).

#### 3.4.3. Effect of Agrobacterium Infection Time and Mode, and Co-Culture Time on Rhizome Transformation Rate

**Figure 6:**
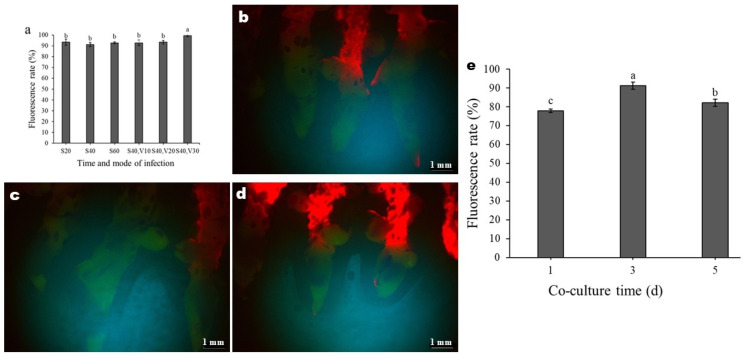
Effects of infection time and mode on fluorescence responses of rhizomes: (**a**) Fluorescence rate at different times and modes of infection. S20, S40, and S60 indicate shaking for 20, 40, and 60 min, respectively. V10, V20, and V30 indicate Vacuum for 10, 20, and 30 min, respectively. (**b**–**d**) Fluorescence response maps of rhizomes after shaking for 20, 40, and 60 min, respectively. The green part of GFP has been successfully expressed, and the red part of GFP has not been expressed. (**e**) Effects of co-culture time on fluorescence rate. Data are shown as mean ± SD. Different letters indicate significant differences at *p* < 0.05.

Agrobacterium infection time, mode, and co-culture time critically influence the infection rate [12,35,36,37,38]. As shown in Figure 6a, the shaking for different times did not significantly affect the fluorescence rate of *C. kanran* ‘Zhushalan’ rhizomes. However, different vacuum times significantly affected the fluorescence rate (Figure 6a–d). Notably, the fluorescence rate was highest following shaking for 40 min, assisted with 30 min of vacuum extraction (Figure 6a). Therefore, combining 40 min of shaking with 30 min of vacuum extraction is optimal for achieving higher transformation efficiency of *C. kanran* ‘Zhushalan’.

Regarding the co-culture time, the fluorescence rate of the rhizomes after three days of co-cultivation was significantly higher than after one or five days (Figure 6e). Therefore, three days was chosen as the optimal co-culture time.

### 3.5. Establishment of Regeneration System for C. kanran ‘Zhushalan’

#### 3.5.1. Selection of the Optimal Dark Culture Time for the Survival of Rhizomes

**Figure 7:**
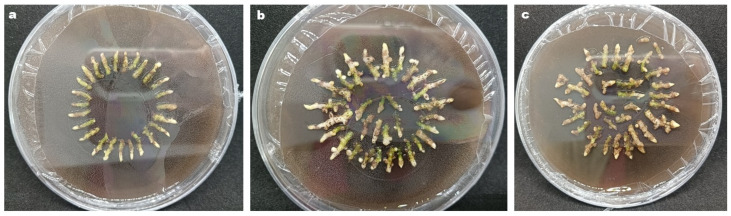
Morphology of regenerated rhizomes after dark culture for: (**a**) 5 days; (**b**) 10 days; (**c**) 15 days.

To determine the effect of dark culture time on the regeneration of *C. kanran* ‘Zhushalan’ rhizomes during genetic transformation, we measured the survival rate and number of regenerated shoots after 30 days of transformation under different dark culture times (Table 5). After 10 days of dark cultivation, the number of surviving rhizomes was significantly higher than that after 5 or 15 days (Table 5). Similarly, the budding number, survival rate, and regeneration frequency recorded after 10 days of dark cultivation were significantly higher than those observed after 5 or 15 days (Table 5). From a growth standpoint, slight browning occurred after 5 and 10 days of dark pre-culture, but it did not affect normal growth (Figure 7). However, when the dark cultivation time increased to 15 days, severe browning of the rhizomes occurred, affecting growth. Thus, 10 days of dark cultivation was determined to be the optimal time for higher regeneration of *C. kanran* ‘Zhushalan’ after transformation.

#### 3.5.2. Selection of the Optimal Bacterial Concentration for Regeneration of ‘Zhushalan’ Rhizomes

The concentration of the Agrobacterium solution significantly impacts the regeneration of transformed plants. As shown in Table 6, our results revealed that both high and low concentrations of the Agrobacterium solution significantly affected the survival and regeneration of ‘Zhushalan’ rhizomes. When the Agrobacterium concentration was OD_600_ = 0.6, the number of surviving rhizomes and regenerated buds was significantly higher than at other concentrations (Table 6). These results indicate that when the concentration of Agrobacterium OD_600_ is 0.6, the regeneration ability of rhizomes is the best. Therefore, to maximize the survival rate and regeneration frequency of transformed ‘Zhushalan’ rhizomes, the Agrobacterium infection solution concentration should be controlled at around OD_600_ = 0.6.

#### 3.5.3. Selection of Optimal Infection Time and Mode for Regeneration of ‘Zhushalan’ Rhizomes

**Figure 8:**
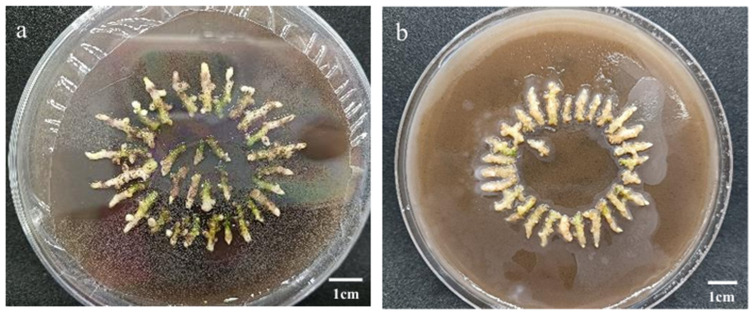
Effect of different times and modes of infection on rhizome regeneration. (**a**) Shaken for 40 min; (**b**) Shaken for 40 min and vacuumed for 30 min.

To investigate the effects of different infection times and methods on the regeneration status of ‘Zhushalan’ rhizomes, we compared the survival and number of regenerated rhizomes under different treatments (Table 7). The results showed that the infection time and methods significantly influenced the survival and regeneration numbers of rhizomes. Vacuum treatment helped to some extent in the genetic transformation of rhizomes. In general, our results showed that the combination of shaking and vacuum treatment significantly decreased the survival rate and regeneration frequency of ‘Zhushalan’ rhizomes (Table 7). Additionally, restoring the culture of rhizomes after vacuum treatment is more difficult than with rhizomes subjected to shaking only (Figure 8).

Of the different shaking treatments, those involving 20 min or 40 min of shaking yielded significantly higher rhizome survival rates than the 60 min treatment (Table 7). However, there was no significant difference in the regeneration frequency among the three treatments (i.e., 20, 40, or 60 min). These results demonstrate that prolonged Agrobacterium infection time can lead to a decrease in the survival rate of rhizomes but has a relatively small impact on regeneration and proliferation. Overall, shaking for 20 to 40 min is more conducive to the recovery and regeneration of ‘Zhushalan’ rhizomes after genetic transformation.

#### 3.5.4. Selection of the Optimal Co-Culture Time for Regeneration of ‘Zhushalan’ Rhizomes

**Figure 9:**
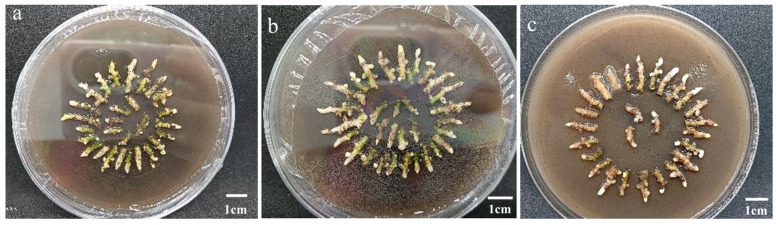
Effect of different co-culture times on rhizome regeneration. (**a**) one day; (**b**) three days; (**c**) five days. The diameter of the Petri dish is 8 cm.

An optimal co-culture time is essential for maximizing the regeneration frequency of rhizomes. As shown in Table 8, our results indicate that co-culturing ‘Zhushalan’ rhizomes with Agrobacterium for too long significantly reduces their survival rate and regeneration frequency. The survival rate and regeneration frequency after one (OND) or three (THD) days of co-culturing were the highest and statistically identical (Table 8). From a growth perspective, the rhizomes co-cultured for five days exhibited severe browning, with significantly inferior growth and regeneration compared to the other two groups (Figure 9). Accordingly, one to three days was selected as the optimal co-culture time for higher regeneration of ‘Zhushalan’ rhizomes after genetic transformation.

### 3.6. Identification of Resistant Plants

**Figure 10:**
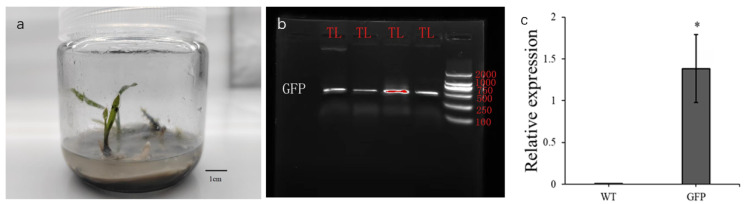
Molecular identification of positive genetically transformed *C. kanran* ‘Zhushalan’ plants. (**a**) Resistant plants of *C. kanran* ‘Zhushalan’; (**b**) PCR detection of positive transgenic plants; (**c**) Relative expression of GFP between wild and transgenic plants. WT and GFP represent wild and transgenic plants, respectively. * indicates significant difference at *p* < 0.05.

First, preliminary transgenic plants with positive results with GFP fluorescence were screened at the early stage of transformation and then further cultivated. After screening with antibiotics, resistant *C. kanran* ‘Zhushalan’ plants were obtained (Figure 10a). Of the 180 transformed *C. kanran* ‘Zhushalan’ rhizomes, 21 resistant plants were obtained. The resistant plants were then used as experimental materials for PCR and qRT-PCR detection. The target bands and differences in the relative expression levels of the GFP gene were detected in all 21 resistant plants (Figure 10b,c). Ultimately, 21 transgenic *C. kanran* ‘Zhushalan’ plants were obtained from 180 rhizomes, resulting in a transformation efficiency of 11.67%.

## 4. Discussion

*C*. *kanran* is one of the seven major Chinese orchids and boasts a long history. Due to its high ornamental value and rich cultural significance, its economic value has been expanding. However, the molecular regulation of many *C*. *kanran* traits, such as leaf color and stress resistance, remains unclear. Furthermore, it is difficult to improve these traits through traditional breeding methods. Therefore, establishing an efficient genetic transformation and regeneration system is crucial to enable functional genomics studies and molecular-assisted breeding of important *C*. *kanran* traits. In this study, we comprehensively examined the effects of dark culture time, bacterial concentration, infection method, infection time, and co-culture time on the transformation and regeneration rates of *C*. *kanran* ‘Zhushalan’ rhizomes. Based on these results, we developed an efficient genetic transformation and regeneration system for *C*. *kanran* ‘Zhushalan’.

The size of explants is crucial for stable transformation. In the case of *C*. *kanran*, the rhizomes are similar to immature embryos, capable of stable proliferation, making them excellent explant materials. If orchid embryos or rhizomes that are too large are used, chimeras may form [28]. In this study, 1–2 cm healthy rhizomes were suitable for *C*. *kanran* genetic transformation and regeneration. However, the achieved transformation efficiency (11.67%) is still relatively low. Thus, further studies using different explants as receptor materials are required to improve the transformation efficiency.

The duration of dark culture significantly impacts both the transformation efficiency and the regenerative capacity of rhizomes. The optimal duration of dark culture varies significantly among different orchid species. For example, *D. officinale* can be successfully transformed without undergoing a pre-culture stage [41], whereas *C*. *sinense* requires at least one month of dark culture before transformation [36]. In this study, the rhizomes of *C*. *kanran* ‘Zhushalan’ developed tender shoot tips after a period of dark culture. If the dark culture duration is too short, Agrobacterium infection becomes difficult, resulting in ineffective transformation or a low transformation rate. Conversely, an excessively long dark culture duration can hinder the complete elimination of Agrobacterium from the rhizomes, ultimately affecting their regeneration. Our findings indicate that a 10-day dark culture period allows for efficient regeneration and achieves a high transformation rate in *C*. *kanran* rhizomes under identical conditions. Moreover, they suggest that moderate dark culture times should be applied for Cymbidium species.

The concentration of Agrobacterium and the duration of infection significantly impact rhizome regeneration and transformation rates. If the concentration of the bacterial solution is too low or the infection duration is too short, Agrobacterium cannot fully adhere to the receptor tissue and fails to achieve effective transformation [42,43]. Conversely, a high bacterial solution concentration or long infection duration can damage the receptor material, further affecting its regeneration [42,43]. We compared the transformation rates of *C*. *kanran* ‘Zhushalan’ rhizomes under different bacterial solution concentrations and infection times and found no significant differences in transformation rates. However, there were significant differences in regeneration frequency. An Agrobacterium infection solution concentration of around OD_600_ = 0.6 with an infection time of 20 to 40 min was suitable to maximize the transformation rate and regeneration frequency of ‘Zhushalan’ rhizomes. This confirms that, when the Agrobacterium solution concentration is low and the infection duration is appropriate, there is no overflow of Agrobacterium on the surface of rhizomes, allowing them to grow normally.

The receptor tissues of Cymbidium species, such as the rhizomes (PLBs), have dense structures that provide substantial physical resistance to natural *Agrobacterium* infection [44]. The exogenous addition of AS effectively induces the expression of virulence (vir) genes on the Agrobacterium Ti plasmid, thereby enhancing infectivity [36,45,46]. This process helps overcome host cell wall barriers and promotes the efficient transfer and integration of T-DNA. In this study, we found that 100 μmol·L^−1^ is the optimal AS concentration for achieving higher transformation efficiency in *C*. *kanran* ‘Zhushalan’. However, this concentration might be more appropriate and specific for the identified Agrobacterium strain EHA105. Therefore, additional investigations are needed before applying this concentration to other Agrobacterium strains or Cymbidium species.

Co-culture time is also an important factor in genetic transformation. Different orchids have different requirements for co-culture time. For example, extending the co-culture time is beneficial for improving the transformation rate in *D*. *nobile* [47]. In contrast, in *P. aphrodite*, extending the co-culture time does not increase the transformation efficiency. Instead, it leads to the death of the explants [10]. In this study, co-culture time had no significant impact on the transformation rate of *C*. *kanran* ‘Zhushalan’. However, when the co-culture time exceeded three days, it became difficult to inhibit Agrobacterium on the surface of the rhizome, resulting in browning and death. Collectively, our findings show that rigorous control of these influencing factors is crucial to efficiently transform *C*. *kanran* ‘Zhushalan’. These factors, among others, including AS concentration, co-culture time, incubation period, antibiotics, dark incubation, humidity, inoculation sites and frequency, *Agrobacterium* strains, and plant genotypes and status, have been revealed to be the most essential in the process of Agrobacterium-mediated genetic transformation of horticultural plants [48].

PCR analysis is an effective method for transgenic detection and is widely used to screen positive transgenic plants. Herein, the band of the target fragment was successfully amplified, confirming the reliability of the transformation system. It is also noteworthy that the concentration of the bacterial solution, infection time and method, and co-culture time had no significant impact on the transformation rate of the *C. kanran* rhizomes. Furthermore, we achieved a transformation efficiency of 11.67% for *C. kanran* ‘Zhushalan’, which is higher than that of *Phalaenopsis* orchid (1.2–5.2%) [17]. However, the achieved transformation efficiency is lower than that in *D. lasianthera* (35–70%) [12], Dendrobium orchids (19.87–27.3%) [30,49], and in Chrysanthemum (*Chrysanthemum* × *morifolium* Ramat.) (51.50%) [50]. The recorded differences in the transformation rate may be attributable to differences in vectors, transformation methods, or explants. The Agrobacterium-mediated genetic transformation system for *C. kanran* established in this study provides a useful tool for functional genomics research and molecular breeding in *Cymbidium*. Further improvement in the established system through evaluating other explants, vectors, and transformation methods, among others, is needed to elevate the transformation rate.

Collectively, this study establishes an efficient Agrobacterium-mediated genetic transformation system for the traditional and highly valued orchid *C. kanran ‘Zhushalan’*. Such a system is an important resource that will enable molecular breeding approaches for key traits that are difficult to achieve through traditional breeding, especially in perennial ornamental plants, where long generation times, limited heritability, and complex crossing can slow trait improvement. With this reliable genetic transformation system, ‘Zhushalan’ can now serve as a chassis for targeted gene function validation and genetic improvement of other Cymbidium species. Breeders and researchers can now directly modify target genes associated with desirable traits and biotic and abiotic stress tolerance. Furthermore, this efficient system may guide the development of a reliable transformation protocol for other orchid plants that are still difficult to transform.

## 5. Conclusions

In this study, we investigated the factors affecting genetic transformation and regeneration of *C*. *kanran* ‘Zhushalan’ and successfully developed an efficient regeneration and genetic transformation system for this orchid. This system exhibited good applicability, achieving a transformation efficiency of 11.67%. Additionally, this efficient Agrobacterium-mediated genetic transformation system holds significant potential for research on functional genes and molecular breeding in *C*. *kanran*. However, further studies on the established system and assessment of other transformation methods are needed to improve the transformation efficiency.

## Figures and Tables

**Table 1:** Type and main components of culture medium used in this study.

Type of Culture Medium	Main Components
Germination medium	1/2MS + 1.0 mg·L^−1^ 6-BA + 1.0 mg·L^−1^ NAA + 1.0 g·L^−1^ AC + 50.0 g·L^−1^ Potato + 80.0 g·L^−1^ Banana
Propagation medium	1/2MS + 2.0 mg·L^−1^ 6-BA + 0.5 mg·L^−1^ NAA + 1.0 g·L^−1^ AC + 50.0 g·L^−1^ Potato + 80.0 g·L^−1^ Banana
Pre-culture medium	1/2MS + 4.0 mg·L^−1^ 6-BA + 0.2 mg·L^−1^ NAA + 1.0 g·L^−1^ AC + 50.0 g·L^−1^ Potato + 80.0 g·L^−1^ Banana
Co-culture medium	1/2MS + 4.0 mg·L^−1^ 6-BA + 0.2 mg·L^−1^ NAA + 100 μmol·L^−1^ AS + 1.0 g·L^−1^ AC + 50.0 g·L^−1^ Potato + 80.0 g·L^−1^ Banana
Regeneration screening medium	1/2MS + 3.0 mg·L^−1^ 6-BA + 2 mg·L^−1^ NAA + 15 mg·L^−1^ Mer + Hyg + 50.0 g·L^−1^ Potato + 80.0 g·L^−1^ Banana

MS: Murashige and Skoog medium; 6-BA: 6-Benzylaminopurine; NAA: 1-naphthlcetic acid; AS: Acetosyringone; Mer: Meropenem; Hyg: Hygromycin. Banana and potato refer to ground fresh potato tubers and bananas in a blender, which were used for medium preparation without filtering.

**Table 2:** Infection capability of different Agrobacterium strains on *C. kanran* ‘Zhushalan’.

Agrobacterium Strain	No. of Rhizomes	No. of GUS Staining	Instantaneous Expression Rate of GUS (%)
LBA4404	30	0.67 ± 1.15 b	2.22 ± 3.85 b
EHA105	30	10.67 ± 0.58 a	35.56 ± 1.92 a
GV3101	30	0.67 ± 0.58 b	2.22 ± 1.92 b

Note: In the same column, different lowercase letters indicate significant differences (*p* < 0.05).

**Table 3:** Effects of different concentrations of acetosyringone on *C. kanran* ‘Zhushalan’ infection.

AS Concentration (μmol·L^−1^)	No. of Rhizomes	No. of GUS Staining	Instantaneous Expression Rate of GUS (%)
0	30	0.00 ± 0.00 c	0.00 ± 0.00 c
100	30	10.33 ± 1.15 a	34.44 ± 3.85 a
200	30	5.33 ± 0.57 b	17.78 ± 1.92 b
300	30	3.33 ± 1.52 b	11.11 ± 5.09 b

Note: In the same column, different lowercase letters indicate significant differences (*p* < 0.05).

**Table 4:** Effects of different concentrations of Hygromycin on the rhizomes of *C. kanran* ‘Zhushalan’.

Concentration of Hgy mg·L^−1^	Number of Dead Rhizomes	Death Frequency of Rhizomes (%)
0	0.00 ± 0.00 a	0.00 ± 0.00 a
20	7.67 ± 1.53 b	25.56 ± 5.09 b
30	13.00 ± 1.00 c	43.33 ± 3.34 c
40	22.67 ± 1.53 d	75.56 ± 5.09 d
50	28.67 ± 1.53 e	95.56 ± 5.09 e
60	30.00 ± 0.00 e	100.00 ± 0.00 e

Values are mean ± SD (n = 3). Means with different letters within a column are significantly different (*p* < 0.05).

**Table 5:** Effects of dark culture time on regeneration of rhizome of *C. kanran* ‘Zhushalan’.

Influence Condition	Processing Method	Number of Rhizomes	Survival Number	Budding Number	Survival Rate (%)	Regeneration Frequency (%)
Dark culture time	FD	60	46.67 ± 3.12 b	42.67 ± 10.69 b	77.78 ± 5.36 b	91.37 ± 17.82 b
	TD	60	59.67 ± 0.58 a	92.67 ± 2.08 a	99.44 ± 0.96 a	155.34 ± 4.98 a
	FTD	60	42.67 ± 2.08 b	36.67 ± 1.53 b	71.11 ± 3.47 b	86.00 ± 3.71 b

FD, five days of dark culture time; TD, ten days of dark culture time; FTD, fifteen days of dark culture time. Values are mean ± SD (n = 3). Means with different letters within a column are significantly different (*p* < 0.05).

**Table 6:** Effects of bacterial concentration on regeneration of *C. kanran* ‘Zhushalan’ rhizomes.

Influence Condition	Processing Method	Number of Rhizomes	Survival Number	Budding Number	Survival Rate (%)	Regeneration Frequency (%)
Bacterial concentration	OD0.4	60	47.67 ± 1.53 b	53.33 ± 1.53 b	79.45 ± 2.54 b	111.93 ± 3.35 b
	OD0.6	60	59.67 ± 0.58 a	81.67 ± 1.53 a	99.44 ± 0.96 a	136.86 ± 1.38 a
	OD0.8	60	36.67 ± 1.53 c	42.33 ± 1.53 c	61.11 ± 2.55 c	115.48 ± 1.83 b
	OD1.0	60	26.67 ± 1.53 d	15.00 ± 1.00 d	44.45 ± 2.55 d	56.42 ± 5.57 c

OD0.4, OD0.6, OD0.8, and OD1.0 indicate OD600 of 0.4, 0.6, 0.8, and 1.0, respectively. Values are mean ± SD (n = 3). Means with different letters within a column are significantly different (*p* < 0.05).

**Table 7:** Effects of different times and modes of infection on the regeneration of *C. kanran* ‘Zhushalan’ rhizomes.

Influence Condition	Processing Method	Number of Rhizomes	Survival Number	Budding Number	Survival Rate (%)	Regeneration Frequency (%)
Timing and mode of infection	S20	60	59.67 ± 0.58 a	91.67 ± 2.08 a	99.44 ± 0.96 a	153.63 ± 2.67 a
	S40	60	59.67 ± 0.58 a	92.67 ± 2.08 a	99.44 ± 0.96 a	155.34 ± 4.99 a
	S60	60	52.33 ± 1.53 b	75.67 ± 1.53 b	87.22 ± 2.54 b	144.61 ± 1.83 a
	S40, V10	60	28.33 ± 1.53 c	15.33 ± 1.53 c	47.22 ± 2.55 c	54.31 ± 7.1 b
	S40, V20	60	17.67 ± 1.15 d	10.67 ± 2.52 cd	29.44 ± 1.93 d	60.47 ± 14.87 b
	S40, V30	60	24.00 ± 1.00 c	10.00 ± 1.00 d	40 + 4.41 c	42.33 + 9.11 d

S20, S40, and S60 indicate shaking for 20, 40, and 60 min, respectively. V10, V20, and V30 indicate Vacuum for 10, 20, and 30 min, respectively. Values are mean ± SD (n = 3). Means with different letters within a column are significantly different (*p* < 0.05).

**Table 8:** Effects of Co-culture time on the regeneration of *C. kanran* ‘Zhushalan’ rhizomes.

Influence Condition	Processing Method	Number of Rhizomes	Survival Number	Budding Number	Survival Rate (%)	Regeneration Frequency (%)
Co-culture time	OND	60	59.33 ± 0.58 a	94.33 ± 3.05 a	98.89 ± 0.96 a	159.00 ± 5.12 a
	THD	60	59.67 ± 0.58 a	92.67 ± 2.08 a	99.44 ± 0.96 a	155.34 ± 4.99 a
	FD	60	44.33 ± 3.51 b	47.00 ± 3.00 b	73.89 ± 5.85 b	106.10 ± 1.69 b

OND, one day of dark Co-culture time; THD, three days of dark Co-culture time; FD, five days of dark Co-culture time. Values are mean ± SD (n = 3). Means with different letters within a column are significantly different (*p* < 0.05).

## Data Availability

The data supporting the reported results are included in this manuscript. The datasets analyzed or generated will be made available on request.

## References

[1] Ovcharenko O.O., Rudas V.A. (2023). Modern Approaches to Genetic Engineering in the Orchidaceae Family. Cytol. Genet..

[2] Iiyama C.M., Vilcherrez-Atoche J.A., Germanà M.A., Vendrame W.A., Cardoso J.C. (2024). Breeding of Ornamental Orchids with Focus on Phalaenopsis: Current Approaches, Tools, and Challenges for This Century. Heredity.

[3] Nirmala C., Nongdam P., Tewari R. (2006). Biotechnological and Molecular Approaches for Improvement of Orchids. Plant Cell Biotechnol. Mol. Biol..

[4] Mehraj H., Raisa R.I., Islam T. (2026). Molecular and Biotechnological Strategies for Improvement of Orchids Using Protocorm-like Bodies: A Comprehensive Review. Next Bioeng..

[5] Zambryski P., Joos H., Genetello C., Leemans J., Van Montagu M., Schell J. (1983). Ti Plasmid Vector for the Introduction of DNA into Plant Cells without Alteration of Their Normal Regeneration Capacity. EMBO J..

[6] Kishi-Kaboshi M., Aida R., Sasaki K. (2018). Genome Engineering in Ornamental Plants: Current Status and Future Prospects. Plant Physiol. Biochem..

[7] Viswanath K.K., Huang J.-Z., Bolaños-Villegas P., Chen F.-C. (2025). Genetic Transformation of Orchids.

[8] Rai G.K., Rai N.P., Kumar S., Yadav A., Rathaur S., Singh M. (2012). Effects of Explant Age, Germination Medium, Pre-Culture Parameters, Inoculation Medium, PH, Washing Medium, and Selection Regime on Agrobacterium-Mediated Transformation of Tomato. Vitr. Cell. Dev. Biol.-Plant.

[9] Song S., Yan R., Wang C., Wang J., Sun H. (2020). Improvement of a Genetic Transformation System and Preliminary Study on the Function of LpABCB21 and LpPILS7 Based on Somatic Embryogenesis in *Lilium pumilum* DC. Fisch. Int. J. Mol. Sci..

[10] Belarmino M.M., Mii M. (2000). Agrobacterium-Mediated Genetic Transformation of a Phalaenopsis Orchid. Plant Cell Rep..

[11] Men S., Ming X., Liu R., Wei C., Li Y. (2003). Agrobacterium-Mediated Genetic Transformation of a Dendrobium Orchid. Plant Cell Tissue Organ Cult..

[12] Utami E.S.W., Hariyanto S., Manuhara Y.S.W. (2018). *Agrobacterium tumefaciens*-Mediated Transformation of *Dendrobium lasianthera* J.J.Sm: An Important Medicinal Orchid. J. Genet. Eng. Biotechnol..

[13] Ratanasut K., Monmai C., Piluk P. (2015). Transient Hairpin RNAi-Induced Silencing in Floral Tissues of Dendrobium Sonia ‘Earsakul’ by Agroinfiltration for Rapid Assay of Flower Colour Modification. Plant Cell. Tissue Organ Cult..

[14] Wang Y., Liu L., Song S., Li Y., Shen L., Yu H. (2017). DOFT and DOFTIP1 Affect Reproductive Development in the Orchid Dendrobium Chao Praya Smile. J. Exp. Bot..

[15] Lee S.H., Li C.W., Liau C.H., Chang P.Y., Liao L.J., Lin C.S., Chan M.T. (2015). Establishment of an Agrobacterium-Mediated Genetic Transformation Procedure for the Experimental Model Orchid Erycina Pusilla. Plant Cell. Tissue Organ Cult..

[16] Thiruvengadam M., Chung I.M., Yang C.H. (2012). Overexpression of Oncidium MADS Box (OMADS1) Gene Promotes Early Flowering in Transgenic Orchid (Oncidium Gower Ramsey). Acta Physiol. Plant..

[17] Hsing H.X., Lin Y.J., Tong C.G., Li M.J., Chen Y.J., Ko S.S. (2016). Efficient and Heritable Transformation of Phalaenopsis Orchids. Bot. Stud..

[18] Huang W., Fang Z., Zeng S., Zhang J., Wu K., Chen Z., da Silva J.A.T., Duan J. (2012). Molecular Cloning and Functional Analysis of Three FLOWERING LOCUS T (FT) Homologous Genes from Chinese Cymbidium. Int. J. Mol. Sci..

[19] Meng N., Liu Y., Dou X., Liu H., Li F. (2018). Transient Gene Expression in Phalaenopsis Aphrodite Petals via *Agrobacterium tumefaciens* Infiltration. Acta Bot. Boreali-Occident. Sin..

[20] Xu Q., Wang S., Hong H., Zhou Y. (2019). Transcriptomic Profiling of the Flower Scent Biosynthesis Pathway of Cymbidium Faberi Rolfe and Functional Characterization of Its Jasmonic Acid Carboxyl Methyltransferase Gene. BMC Genom..

[21] Yu Z., Zhao C., Zhang G., Teixeira da Silva J.A., Duan J. (2020). Genome-Wide Identification and Expression Profile of TPS Gene Family in Dendrobium Officinale and the Role of DoTPS10 in Linalool Biosynthesis. Int. J. Mol. Sci..

[22] Liu J.X., Chiou C.Y., Shen C.H., Chen P.J., Liu Y.C., Der Jian C., Shen X.L., Shen F.Q., Yeh K.W. (2014). RNA Interference-Based Gene Silencing of Phytoene Synthase Impairs Growth, Carotenoids, and Plastid Phenotype in Oncidium Hybrid Orchid. Springerplus.

[23] Yeh C.W., Liu J., Chiou C.Y., Shen C.H., Chen P.J., Liou J.C., Der Jian C., Shen X.L., Shen F.Q., Yeh K.W. (2017). Functional Study of Phytoene Synthase by RNAi-Based Downregulation in the Oncidesa Orchid. Orchid Biotechnol. III.

[24] Chang L., Chang H.-H., Chiu Y.-S., Chang J.-C., Hsu D.-W., Tzean Y., Cheng A.-P., Lu H.-C., Yeh H.-H. (2019). Plant A20/AN1 Proteins Coordinate Different Immune Responses Including RNAi Pathway for Antiviral Immunity. bioRxiv.

[25] Chao Y.T., Chen W.C., Chen C.Y., Ho H.Y., Yeh C.H., Kuo Y.T., Su C.L., Yen S.H., Hsueh H.Y., Yeh J.H. (2018). Chromosome-Level Assembly, Genetic and Physical Mapping of Phalaenopsis Aphrodite Genome Provides New Insights into Species Adaptation and Resources for Orchid Breeding. Plant Biotechnol. J..

[26] Chung O., Kim J., Bolser D., Kim H.M., Jun J.H., Choi J.P., Do Jang H., Cho Y.S., Bhak J., Kwak M. (2022). A Chromosome-Scale Genome Assembly and Annotation of the Spring Orchid (*Cymbidium goeringii*). Mol. Ecol. Resour..

[27] Ai Y., Li Z., Sun W.H., Chen J., Zhang D., Ma L., Zhang Q.H., Chen M.K., Zheng Q.D., Liu J.F. (2021). The Cymbidium Genome Reveals the Evolution of Unique Morphological Traits. Hortic. Res..

[28] Kuehnle A.R., Sugii N. (1992). Transformation of Dendrobium Orchid Using Particle Bombardment of Protocorms. Plant Cell Rep..

[29] Uddain J., Zakaria L., Lynn C.B., Subramaniam S. (2015). Preliminary Assessment on Agrobacterium-Mediated Transformation of Dendrobium Broga Giant Orchid’s PLBs. Emir. J. Food Agric..

[30] Phlaetita W., Chin D.P., Otang N.V., Nakamura I., Mii M. (2015). High Efficiency Agrobacterium-Mediated Transformation of Dendrobium Orchid Using Protocorms as a Target Material. Plant Biotechnol..

[31] Niyomtham K., Bhinija K., Huehne P.S. (2018). A Direct Gene Transferring System for Oncidium Orchids, a Difficult Crop for Genetic Transformation. Agric. Nat. Resour..

[32] Griesbach R.J., Hammond J. (1993). Incorporation of the Gus Gene into Orchids Through Embryo Electrophoresis. Acta Hortic..

[33] Teixeira da Silva J.A., Aceto S., Liu W., Yu H., Kanno A. (2014). Genetic Control of Flower Development, Color and Senescence of Dendrobium Orchids. Sci. Hortic..

[34] Mii M., Chin D.P. (2018). Genetic Transformation on Orchid Species: An Overview of Approaches and Methodologies.

[35] Ruo-jun M. (2008). Preliminary Study on the Genetic Transformation Systems of Phalaenopsis and Oncidium. J. Anhui Agric. Sci..

[36] Xie L., Wang F., Zeng R., Guo H., Zhou Y., Zhang Z. (2015). Agrobacterium-Mediated Transformation of Cymbidium Sinensis. Shengwu Gongcheng Xuebao/Chin. J. Biotechnol..

[37] Nopitasari S., Setiawati Y., Lawrie M.D., Purwantoro A., Widada J., Sasongko A.B., Yoshioka Y., Matsumoto S., Ninomiya K., Asano Y. (2020). Development of an Agrobacterium-Delivered Crispr/Cas9 for *Phalaenopsis Amabilis* (L.) Blume Genome Editing System. AIP Conf. Proc..

[38] Li X.Q., Sheng Y.H., Fu Y.G., Zhou Z., Zhao Y., Lin P., Song X.Q., Wang J. (2020). Establishment of Efficient Regeneration System of Oncidium. J. South. Agric..

[39] Liu Y.-S., Wang H.-Y., Zhao Y.-J., Jin Y.-B., Li C., Ma F.-W. (2022). Establishment of an Efficient Regeneration and Genetic Transformation System for Malus Prunifolia Borkh. ‘Fupingqiuzi’. J. Integr. Agric..

[40] Chen J., Wang L., Chen J., Huang J., Liu F., Guo R., Yang L., Grabon A., Zhao K., Kong F. (2018). *Agrobacterium tumefaciens*-Mediated Transformation System for the Important Medicinal Plant Dendrobium Catenatum Lindl. Vitr. Cell. Dev. Biol.-Plant.

[41] Kui L., Chen H., Zhang W., He S., Xiong Z., Zhang Y., Yan L., Zhong C., He F., Chen J. (2017). Building a Genetic Manipulation Tool Box for Orchid Biology: Identification of Constitutive Promoters and Application of CRISPR/Cas9 in the Orchid, Dendrobium Officinale. Front. Plant Sci..

[42] Pushyami B., Beena M.R., Sinha M.K., Kirti P.B. (2011). In Vitro Regeneration and Optimization of Conditions for Agrobacterium Mediated Transformation in Jute, Corchorus Capsularis. J. Plant Biochem. Biotechnol..

[43] Yan R., Wang Z., Ren Y., Li H., Liu N., Sun H. (2019). Establishment of Efficient Genetic Transformation Systems and Application of CRISPR/Cas9 Genome Editing Technology in Lilium Pumilum DC. Fisch. and Lilium Longiflorum White Heaven. Int. J. Mol. Sci..

[44] Stachel S.E., Messens E., Van Montagu M., Zambryski P. (1985). Identification of the Signal Molecules Produced by Wounded Plant Cells That Activate T-DNA Transfer in *Agrobacterium tumefaciens*. Nature.

[45] Yang J., Lee H.J., Shin D.H., Oh S.K., Seon J.H., Paek K.Y., Han K.H. (1999). Genetic Transformation of Cymbidium Orchid by Particle Bombardment. Plant Cell Rep..

[46] Nayak N.R., Tanaka M., Teixeira daSilva J.A. (2006). Biotechnology of Cymbidium—An Overview of Recent Progress and Future Opportunities. Floric. Ornam. Plant Biotechnol. Adv. Top. Issues.

[47] Wang H., Deng N., Zhang Y., Liang S. (2019). The Establishment of Genetically Modified Dendrobium Nobile Tissues. J. South China Norm. Univ. (Nat. Sci. Ed.).

[48] Hao S., Zhang Y., Li R., Qu P., Cheng C. (2024). Agrobacterium-Mediated in Planta Transformation of Horticultural Plants: Current Status and Future Prospects. Sci. Hortic..

[49] Suwanaketchanatit C., Piluek J., Peyachoknagul S., Huehne P.S. (2007). High Efficiency of Stable Genetic Transformation in Dendrobium via Microprojectile Bombardment. Biol. Plant..

[50] Li Y., Lu M., Wang J., Deng C., Lu C., Cui Y., Tian Y., Feng B., Hong Y., Dai S. (2026). Establishment of a High-Efficiency Protoplast Isolation and Transient Transformation System for Chrysanthemum Petals. Horticulturae.

